# Loss of *Col3a1*, the Gene for Ehlers-Danlos Syndrome Type IV, Results in Neocortical Dyslamination

**DOI:** 10.1371/journal.pone.0029767

**Published:** 2012-01-03

**Authors:** Sung-Jin Jeong, Shihong Li, Rong Luo, Natalie Strokes, Xianhua Piao

**Affiliations:** Division of Newborn Medicine, Department of Medicine, Children's Hospital and Harvard Medical School, Boston, Massachusetts, United States of America; Medical College of Georgia, United States of America

## Abstract

It has recently been discovered that Collagen III, the encoded protein of the type IV Ehlers-Danlos Syndrome (EDS) gene, is one of the major constituents of the pial basement membrane (BM) and serves as the ligand for GPR56. Mutations in *GPR56* cause a severe human brain malformation called bilateral frontoparietal polymicrogyria, in which neurons transmigrate through the BM causing severe mental retardation and frequent seizures. To further characterize the brain phenotype of *Col3a1* knockout mice, we performed a detailed histological analysis. We observed a cobblestone-like cortical malformation, with BM breakdown and marginal zone heterotopias in *Col3a1* ^−/−^ mouse brains. Surprisingly, the pial BM appeared intact at early stages of development but starting as early as embryonic day (E) 11.5, prominent BM defects were observed and accompanied by neuronal overmigration. Although collagen III is expressed in meningeal fibroblasts (MFs), *Col3a1* ^−/−^ MFs present no obvious defects. Furthermore, the expression and posttranslational modification of α-dystroglycan was undisturbed in *Col3a1* ^−/−^ mice. Based on the previous finding that mutations in *COL3A1* cause type IV EDS, our study indicates a possible common pathological pathway linking connective tissue diseases and brain malformations.

## Introduction

Cortical dyslamination is an important cause of neurological morbidity. Cobblestone lissencephaly is one common form of cortical dyslamination, in which neurons migrate beyond the breached pial BM and form ectopias on the surface of the brain [1]. Cobblestone lissencephaly is seen in three types of human congenital muscular dystrophy syndromes; Walker -Warburg syndrome (WWS), Fukuyama-type muscular dystrophy (FCMD), and muscle-eye-brain disease (MEB). WWS is the most severe form of congenital muscular dystrophy, with the vast majority of patients dying in utero or in early infancy. The genetic cause for MEB, FCMD, and some WWS cases is aberrant glycosylation of α-dystroglycan, a receptor for laminin [2].

GPR56 is a member of the adhesion G protein-coupled receptor (GPCR) family. Mutations in *GPR56* cause a specific human brain malformation called bilateral frontoparietal polymicrogyria (BFPP) [3]–[6]. The magnetic resonance images of BFPP brains revealed a thickened cerebral cortex with coarse gyri, shallow sulci, and a “scalloped” appearance at the grey-white matter junction – much like the radiological features of other polymicrogyria malformations. Histological analysis of *Gpr56* knockout mouse brains and postmortem human BFPP brains revealed the histopathology of BFPP to be cobblestone lissencephaly [7], [8].

Collagen III is a major collagen found in connective tissues. Mutations in one allele of *COL3A1* cause type IV EDS, an autosomal dominant connective tissue disorder [9]–[14]. Recently, we discovered that collagen III is the ligand of GPR56 [15]. In this paper, we carried out a detailed histological analysis of *Col3a1* ^−/−^ mouse brains. We found that the absence of collagen III results in a cobblestone-like cortical malformation.

## Results

### Cobblestone-like cortical malformation is associated with homozygous deletion of *Col3a1*

Although losing one allele of the *Col3a1* gene is not associated with any obvious defects in mice, the effects of deleting both alleles is catastrophic [16]. *Col3a1* ^−/−^ usually results in perinatal lethality of an unknown etiology, with only 5% of mice reaching adulthood [16]. As for the surviving mice, their phenotype closely resembles the clinical manifestations of patients with type IV EDS, including the rupture of large blood vessels [16]. Due to the severity of this corresponding condition, the *Col3a1* ^−/−^ adult mice experience a signficantly shortened lifespan.

**Figure 1:**
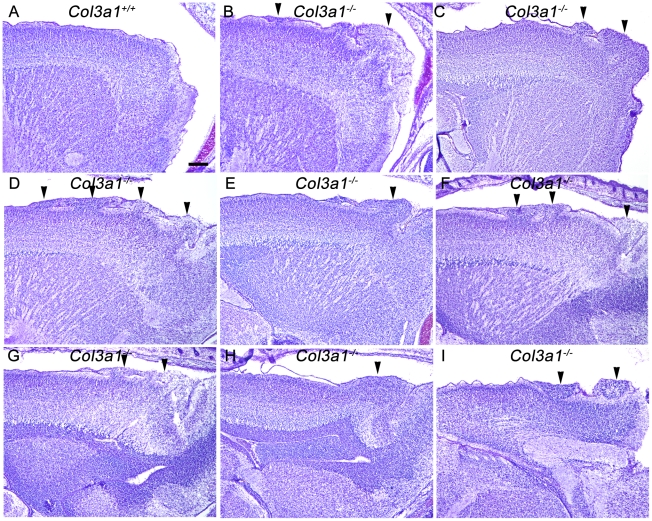
*Col3a1^−/−^* mice have cortical abnormalities. Sagittal sections from one *Col3a1^+/+^* (**A**) and three *Col3a1* ^−/−^ forebrains (**B–I**) stained with Nissl. In contrast to the well-developed cortex in *Col3a1^+/+^* brains (A), cortical malformation was seen in *Col3a1* ^−/−^ brains, characterized by the presence of ectopic clusters of neurons migrating into the marginal zone and disrupting the lamination of the cortex (B–I, arrowheads). Scale bar, 200 µm.

**Figure 2:**
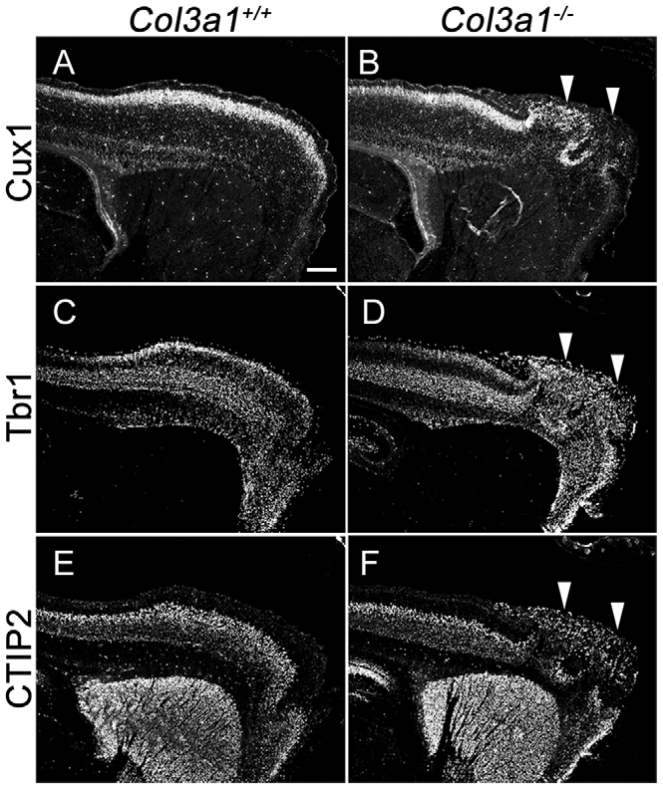
Cellular composition of *Col3a1* ^−/−^ cortical ectopias. (**A, B**) Cux1 antibody immunostaining revealed that the ectopias were composed in part by upper layer cortical neurons inappropriately migrating through the marginal zone in *Col3a1* ^−/−^ brain (arrowheads). (**C, D**) Tbr1 immunostaining revealed that the deeper layer cortical neurons were also present in the ectopias of *Col3a1* ^−/−^ (arrowheads). (**E, F**) Layer V cortical neurons, revealed by CTIP2 immunostaining, that are normally localized to a discrete interior strip of cells were mislocalized in the ectopias of *Col3a1* ^−/−^ (arrowheads). Scale bar, 200 µm.

After discovering that collagen III serves as the major ligand of GPR56, we sought out to investigate the uncharacterized brain phenotype of *Col3a1* ^−/−^ mice. In order to discern the architecture of the cerebral cortex, we first performed Nissl stainings with a cresyl violet solution on the brains of E18.5 mice. All *Col3a1^−/−^* mice showed severe cortical malformation, manifested by the presence of neuronal ectopias on the brain surface (Figure 1 B–I). We next performed immunohistochemistry (IHC) using different layer markers to determine their neuronal composition and from where they first originated. Since over 95% of *Col3a1* ^−/−^ mice were lethal upon birth, we performed immunostainings on E18.5 brains [16]. We used three cortical layer-specific markers, Cux1 for layers II–IV, Tbr1 for layers II–III and VI and CTIP2 for layer V [17]–[19]. Neurons positive for Cux1, Tbr1 and CTIP2 were detected in the ectopias, suggesting that the ectopic cells in the *Col3a1* ^−/−^ cortex were neurons from both deep and superficial cortical layers, mirroring our observations of *Gpr56* null mutant mice (Figure 2B, D and F) [8].

### The pial BM is properly formed but is subsequently disrupted in the *Col3a1* ^−/−^ mouse neocortex

**Figure 3:**
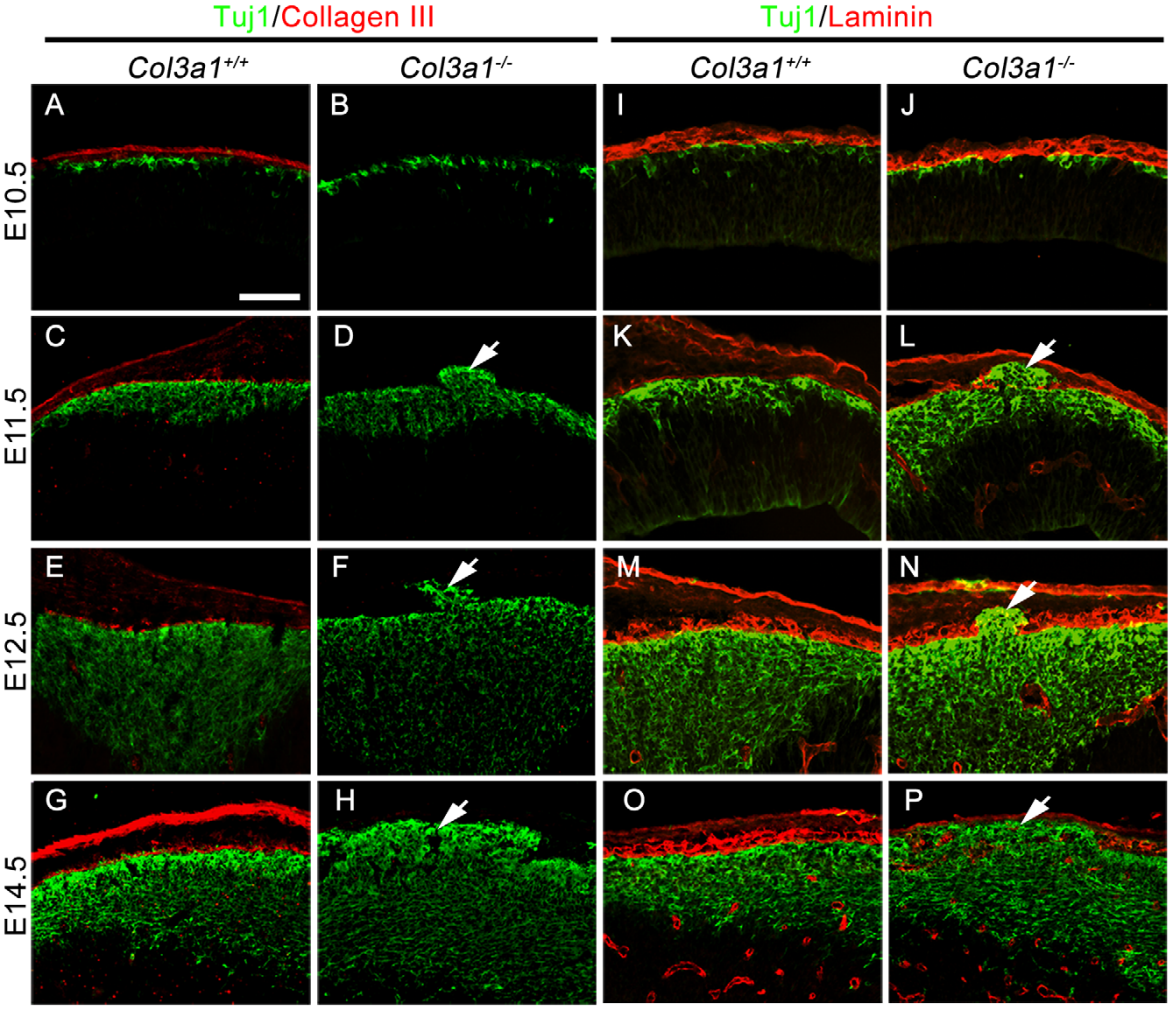
The pial BM is well formed at E10.5 but subsequently disrupted in the *Col3a1^−/−^* neocortex. (**A–H**) Double IHC of Tuj1 and Collagen III in E10.5, E11.5, E12.5, and E14.5 brains. Collagen III was absent in all analyzed brains of *Col3a1^−/−^* mice. Tuj1^+^ migrating neurons (green) were well organized beneath the pial BM (red) in both *Col3a1* ^+/+^ and *Col3a1^−/−^* at E10.5 (A and B), whereas Tuj1^+^migrating neurons (green) migrated past the pial BM into the arachnoid space (arrow) in the brains of *Col3a1^−/−^* mice at E11.5 and older (D, F, and H). (**I–P**) Double IHC of Tuj1 and laminin in E10.5, E11.5, E12.5, and E14.5 mouse brains. Tuj1^+^ neurons (green) were properly localized beneath the pial BM (red) in the brains of *Col3a1^+/+^* mice at all embryonic days analyzed (I, K, M and O) and *Col3a1^−/−^* mouse at E10.5 (J). In contrast, ectopias were observed in the brains of *Col3a1^−/−^* mice from E11.5 through E14.5 (arrows, L, N, and P). Scale bar, 100 µm.

To identify the leading pathology associated with *Col3a1* deletion, we performed a detailed time course study of the occurrence of the breached pial BM and overmigrated neurons. While collagen III was expressed in the meninges and pial BM of *Col3a1* ^+/+^ brains (Figure 3A, C, E, and G), the *Col3a1^−/−^* mice appeared to be true deletion mutants since collagen III was not present in either the meninges or the pial BM in brains ranging from E10.5–E14.5 (Figure 3B, D, F, and H). Interestingly, in spite of the absence of collagen III, the pial BM was initially properly formed at E10.5 in the mutant mice (Figure 3J). Regional breakdown of the pial BM with concurrent neuronal overmigration was observed in about half of the E11.5 and all embryos older than E12.5 in the *Col3a1* ^−/−^ brains analyzed (arrows, Figure 3L, N, P and Table 1).

**Table 1:** Penetrance of cortical dysplasia in *Col3a1* mice.

Stage	No. of animals with ectopia/No. of total animals analyzed		
	Wild-type	Heterozygous	Null
E10.5	0/2	0/1	0/3
E11.5	0/2	0/1	3/5
E12.5	0/4	0/5	5/5
E14.5	0/4	0/2	4/4
E16.5	0/3	0/1	4/4
E18.5	0/5	0/2	7/7
Summary	0/20	0/12	23/28

### Deleting *Col3a1* results in abnormal attachment of radial glial endfeet

**Figure 4:**
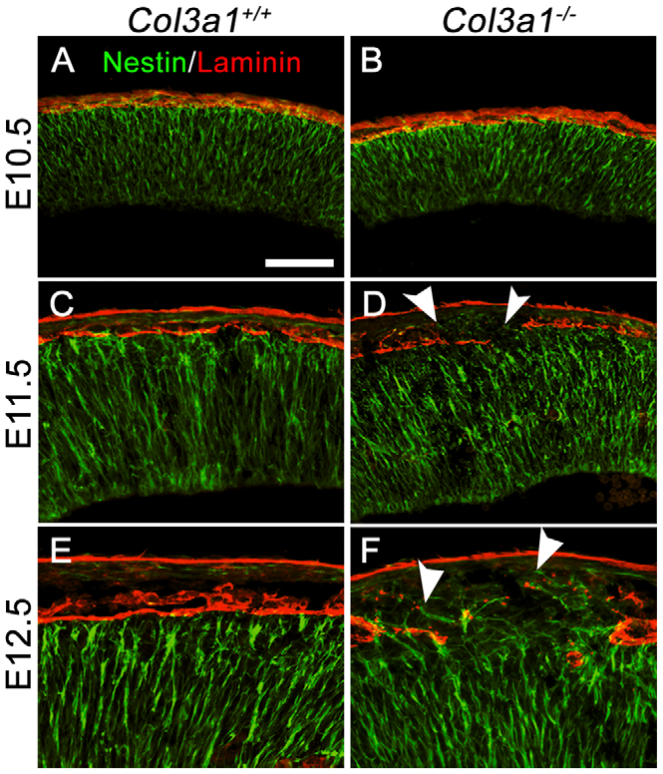
Radial glial endfeet protrude into the ectopias of *Col3a1^−/−^* mice. (**A** and **B**) Double IHC of nestin (green) and laminin (red) at E10.5 showed a parallel arrangement of fibers that terminated in well defined endfeet at the pial surface in both *Col3a1* ^+/+^ (A) and *Col3a1^−/−^* mice (B). (**C–F**) Nestin^+^ radial glial endfeet (green) lined up nicely along the pial BM (red) in E11.5 and E12.5 *Col3a1* ^+/+^ mice (C and E) but were abnormally located in the arachnoid space of ectopias in the region of breached pial BM (arrowheads) in *Col3a1^−/−^* mice (D and F). Scale bar, 100 µm.

During normal brain development, radial glial endfeet attach to the pial BM and form an adhesive lining at the pial surface [20]. Since the proper attachment of the radial glial endfeet is relavent to the integrity of the pial BM, we therefore examined the arrangement of the endfeet in relationship to the pial BM by double IHC of nestin and laminin. At E10.5, radial glial endfeet were arranged in an orderly fashion along the intact pial BM in the brains of both *Col3a1* ^+/+^ and *Col3a1* ^−/−^ mice (Figure 4A and B). We observed protruded endfeet through a breached pial BM in some of the E11.5 and all of the E12.5 *Col3a1* ^−/−^ brains (arrowheads, Figure 4D and F).

### Loss of collagen III leads to abnormal positioning of both Cajal-Retzius (CR) cells and interneurons

**Figure 5:**
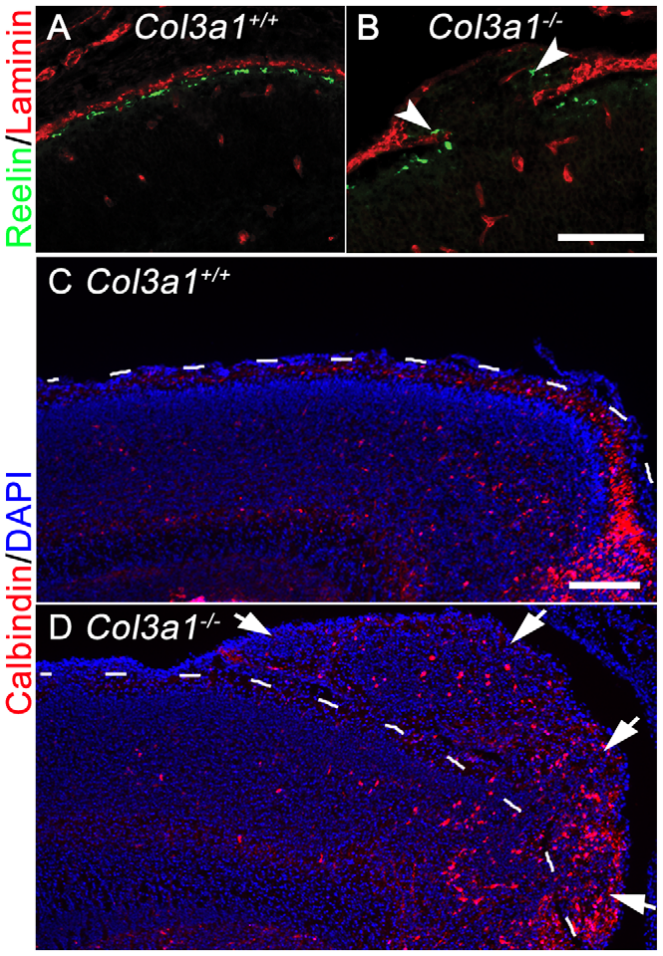
Cajal-Retzius cells and interneurons are found in ectopias of *Col3a1^−/−^* mice. (**A** and **B**) Double IHC of Reelin (green) and laminin (red) at E16.5 showed Reelin^+^ CR cells are lined up beneath the pial BM in *Col3a1* ^+/+^ (A) but were located within the ectopia of *Col3a1^−/−^* mice (B, arrowheads). (**C** and **D**) Immunostaining of Calbindin (red) at E18.5. Calbindin^+^ interneurons are observed in the ectopias of *Col3a1^−/−^* mice (D, arrows) but were normally localized within the marginal zone and cortical plate in *Col3a1* ^+/+^ brain (C). The pia surface of the brains is outlined in white. Nuclear counterstain was performed by Hoechst 33342 (blue). Scale bars, A and B, 100 µm; C and D, 200 µm.

There are two major neurons in the marginal zone of the developing neocortex – CR cells and interneurons. CR cells regulate the proper positioning of postmitotic neurons during cortical development by secreting reelin, an extracellular matrix (ECM) signaling molecule [21], [22]. To determine whether CR cells are abnormally located in the developing *Col3a1* ^−/−^ cortex, we compared the distribution of CR cells in brains of *Col3a1* ^+/+^ and *Col3a1* ^−/−^ animals using reelin to identify CR cells. In contrast to the relatively continuous single layer of CR cells found at the marginal zone of E16.5 control animals (Figure 5A), we observed misplaced CR cells beyond the defective BM in *Col3a1* ^−/−^ brains (Figure 5B, arrowheads).

In order to investigate whether the migration of interneurons is affected by the loss of collagen III, we performed an IHC of calbindin in E18.5 brains of *Col3a1* wild type and mutant mice. Calbindin^+^ interneurons were well organized beneath the pial BM in *Col3a1^+/+^* brain (Figure 5C). In contrast, we detected calbindin^+^ interneurons in the ectopias of *Col3a1^−/−^* brains (Figure 5D, arrows).

### MFs develop normally in the absence of collagen III

**Figure 6:**
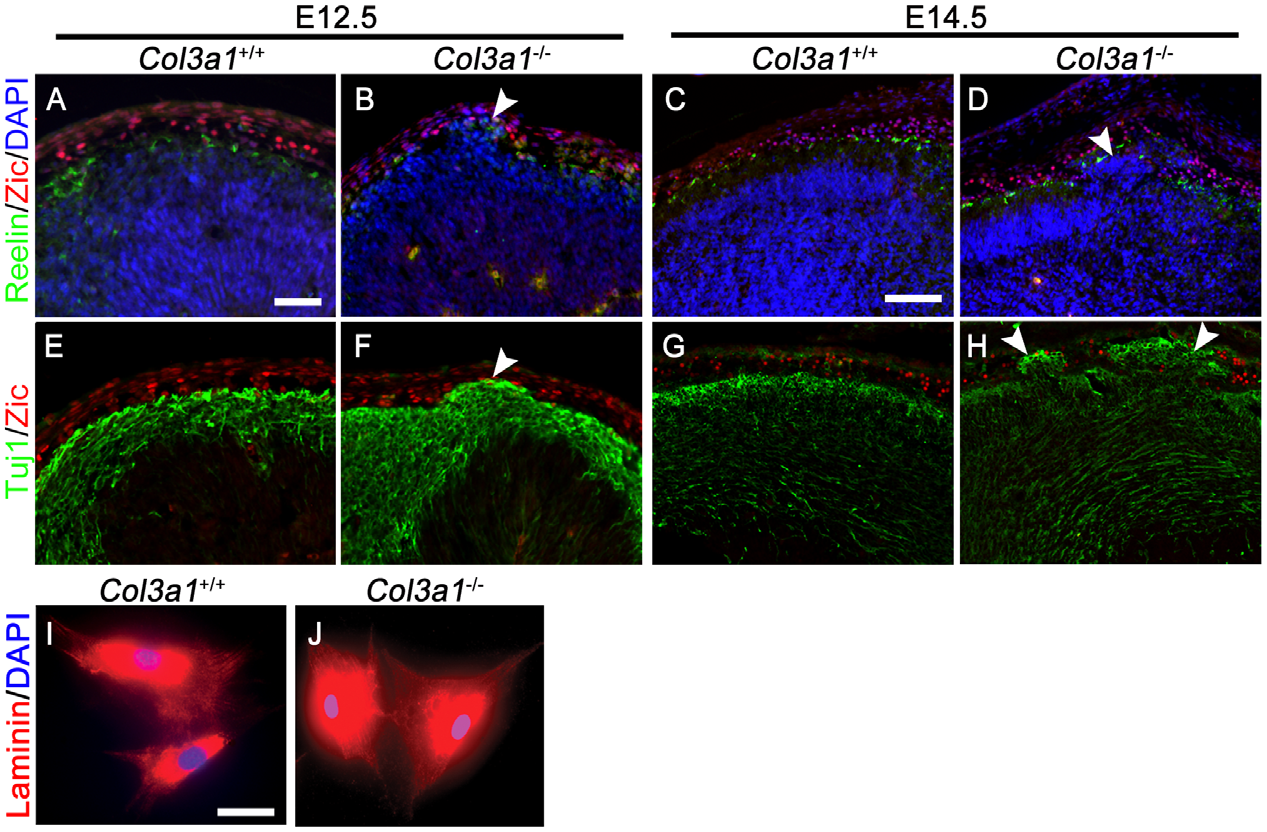
The MFs develop normally in *Col3a1* mutant mice. Double IHC of Zic (red) and Reelin (green) (**A–D**) or Tuj1 (green) (**E–H**) in *Col3a1* ^+/+^ and *Col3a1^−/−^* brains revealed a normal distribution of Zic^+^ cells in all brains analyzed. (**I** and **J**) Laminin staining of primary MFs revealed no differences between *Col3a1^+/+^* and *Col3a1^−/−^* mice. Nuclear counterstaining was performed by Hoechst 33342 (blue). Scale bars, A, B, E and F, I and J, 50 µm; C, D, G and H, 100 µm.

MFs are essential for basal lamina organization and cortical development, as defects in their proliferation and differentiation lead to cobblestone-like cortical malformation [23]–[25]. To study whether the loss of collagen III affects MF development, we studied the distribution of MFs in the *Col3a1* ^−/−^ mouse neocortex using a pan-Zic antibody that recognizes all Zic protein family members. Zic proteins are expressed in both MFs and CR cells at the surface of the developing brain [23]. To specifically reveal the status of MFs, we performed a double IHC of Zic with either reelin and Tuj1. The distribution of Zic^+^ cells in the meninges was comparable between *Col3a1* ^+/+^ and *Col3a1* ^−/−^ brains in all stages evaluated, including the regions with neuronal ectopias (Figure 6A–H). To further examine the quality of *Col3a1* ^−/−^ MFs, we established a primary MF culture from the meninges of *Col3a1* ^+/+^ and *Col3a1* ^−/−^ of E14.5 mice. The cell morphology and the pattern of laminin immunostaining were identical between *Col3a1* ^+/+^ and *Col3a1* ^−/−^ MFs (Figure 6I and J).

### α-Dystroglycan is not affected by *Col3a1* deletion

**Figure 7:**
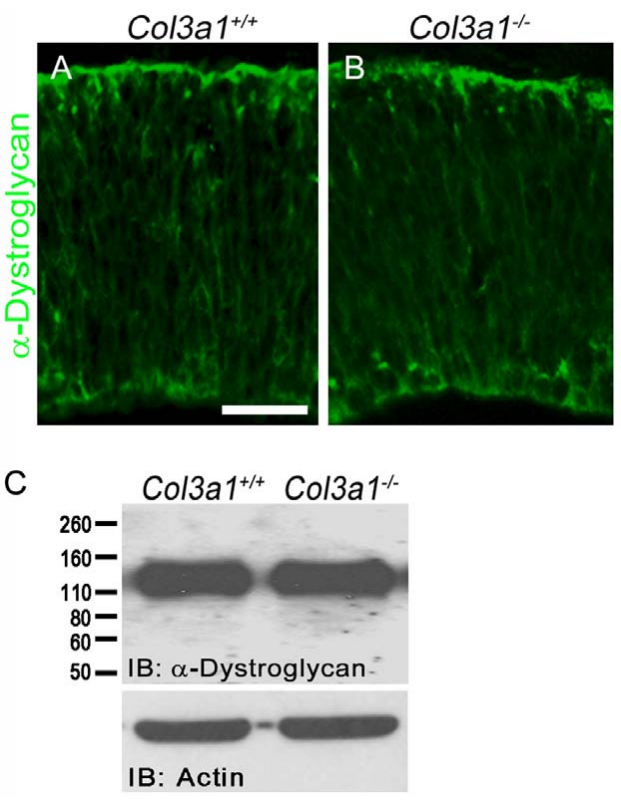
The expression of glycosylated α-Dystroglycan is not affected by loss of collagen III. The level of glycosylated α-Dystroglycan revealed no change between *Col3a1* ^+/+^ and *Col3a1* ^−/−^ E12.5 brains by IHC (**A–B**) and western blot analysis (**C**). Antibody IIH6C4 recognizes the glycosylated form of α-Dystroglycan. Scale bar, 50 µm.

Aberrant glycosylation of α-dystroglycan causes human cobblestone lissencephaly, whereas deleting the mouse *Dag1* gene results in early embryonic lethality [1], [26], [27]. To investigate whether the signaling of GPR56 affects the expression and/or glycosylation status in the mouse developing brain, we performed IHC and western blot analysis with a monoclonal antibody that specifically detects the glycosylated form of α-dystroglycan in *Col3a1* wild type and mutant mouse brains [28]. We failed to detect any change in the level of α-dystroglycan in the brains of *Col3a1* ^−/−^ mice, arguing that the function of collagen III does not directly affect the expression and glycosylation of dystroglycan (Figure 7).

## Discussion

We have shown that homozygous deletion of *Col3a1* causes cobblestone-like cortical malformation characterized by pial BM breakdown, neuronal overmigration, radial glial detachment, and formation of marginal zone heterotopias. While the pial BM is established in the absence of collagen III, focal breaks of the pial BM with concurrent neuronal overmigration become obvious in later embryonic development.

In humans, cobblestone lissencephaly is typically seen in three types of congenital muscular dystrophy, namely WWS, MEB, and FCMD [1]. Although aberrant glycosylation of α-dystroglycan is the leading pathology of human cobblestone lissencephaly, we failed to detect any changes in the level of glycosylated α-dystroglycan in *Col3a1* ^−/−^ [26], [27]. This finding suggests that collagen III regulates cortical development independent of the dystroglycan pathway. Recent findings that mutations in *COL4A1* cause an ocular/muscular/cortical developmental disorder in mice and WWS in humans without affecting the level of glycosylated α-dystroglycan further supports the heterogeneous etiology of cobblestone lissencephaly [29].

Mutant mice with deletions in some members of the integrin family as well as downstream associates of integrins, such as focal adhesion kinase (FAK) and integrin-linked kinase (Ilk) also show cortical migration defects with deficiencies in basal lamina integrity with features that resemble human cobblestone lissencephaly [30]–[37]. Moreover, it has been shown that GPR56 associates with tetraspanins CD9 and CD81 [38]. The function of this tetraspanin-GPR56 complex remains unclear. Members of the tetraspanin family of cell surface proteins act as molecular scaffolds with known adhesion proteins such as integrins to facilitate their function [39]. It is an intriguing question of whether the receptor-ligand pair of GPR56 and collagen III functions together with integrins in regulating cortical development.

On the surface of the brain lies the three layered meninges – the pia, the arachnoid, and the dura – in which the major cell type is MFs. It has been shown that cellular defects in MFs cause abnormal development of structures adjacent to the meninges, which are the skull and the brain. We have recently discovered that collagen III is expressed in abundance in the MFs [15]. However, we detected no obvious defects in the MFs of the *Col3a1* ^−/−^ mice, suggesting that the cortical dyslamination seen in *Col3a1* ^−/−^ mice is not the direct result of cellular defects of MFs, but rather the absence of collagen III, the ligand of GPR56.

EDS is a heterogeneous group of hereditary connective tissue disorders. Individuals with EDS present with joint and skin hyperextensibility and vascular problems, including aortic dissection and excessive bleeding [10]–[14]. There has been a reported association of EDS and periventricular heterotopia, which is characterized by the presence of nodules of neurons along the periventricular region of the brain [40]. Most reported cases of type IV EDS are associated with mutations in one allele of *COL3A1* [14]. However, there is one reported case of recessive type IV EDS with homozygous mutation in *COL3A1* gene and a diffuse cortical dysplasia, which was most prominent frontally [41]. We showed here that homozygous deletion of mouse *Col3a1* results in perinatal lethality and cobblestone-like cortical malformation. It is possible that mutations in both alleles of *COL3A1* associate with a lethal form of cobblestone lissencephaly similar to WWS.

Regulation of pial BM development and remodeling is likely to be dynamic and complex. The pial BM consists of thin sheets of proteins including laminins, collagen IV, nidogens, and perlecan. Collagen III is a type of fibrillar collagen that is thought to be mainly in the ECM of the skin, cardiac, and vascular tissues [42]–[45]. Although there is little knowledge of the presence of collagen III in the developing brain, our recent work confirmed the presence of collagen III in the pial BM by IHC and immunoelectron microscopy [15]. In this report, we revealed the indispensible function of collagen III in cortical development, setting the stage for further mechanistic study of how collagen III regulates brain development.

## Materials and Methods

### Ethics statement

Experiments were performed in accordance with National Institutes of Health guidelines for the care and use of laboratory animals, and with approval of the Animal Care and Use Committee of Children's Hospital Boston (approval ID: A3303-01).

### Antibodies

The antibodies used in the study are peroxidase-conjugated goat anti-mouse IgG antibody (Sigma), rabbit anti-Englebreth-Holm-Swarm laminin (Sigma), rabbit anti-reelin (Chemicon International), mouse anti-Zic (gift from Dr. R. Segal), mouse and rabbit anti-Tuj1 (Covance), rabbit anti-human collagen III (Lifespan Biosciences), rabbit anti-calbindin (Swant), mouse anti-α-dystroglycan, II6C4 (Millipore), rabbit cux1 (a gift from C.A. Walsh, Children's Hospital Boston), rabbit anti-Tbr1 (a gift from R. Hevner, Seattle Children's Research Institute), rat anti-CTIP2 (Abcam), and mouse anti-nestin (BD Transduction Laboratories).

### Mice

*Col3a1* mice were obtained from the Jackson Laboratory with the strain name C.129S4(B6)-*Col3a1^tm1Jae^*/J in a BALB/c background as described previously [16]. Most of the homozygous mutant mice die at birth with only about 5% of them surviving to adulthood [16]. All breeding was carried out with heterozygote crossing.

### Histology and immunohistochemistry (IHC)

Histology analysis was carried out as previously described [8], . Brains harvested from embryos were fixed using 4% paraformaldehyde and were cryoprotected by 30% sucrose. Brain sections obtained by cryostat were stained with 0.1% cresyl violet/0.5% acetic acid for Nissl staining. Sections were processed for immunostaining using standard procedures. Primary antibodies were visualized by appropriate fluorophore-conjugated secondary antibodies. Nuclei were stained with Hoechst 33342 (Invitrogen, 1∶2000). Images were captured using a Nikon 80i upright microscope. Representative photographs were obtained with the same exposure setting for control and mutant.

### Preparation of mouse primary MFs and immunocytochemistry

Mouse primary MFs were established from the meninges of E14.5 *Col3a1* wild type or mutant mice and amplified in DMEM with 10% FBS. MFs were cultured on poly-D-lysine (100 µg/ml) coated wells for 24 hours, followed by fixation with 4% paraformaldehyde. Cells were permeabilized with 0.1% Triton-X 100 in PBS for 10 minutes followed by incubation with anti-laminin antibody and visualized by goat anti-rabbit Alexa-Fluor 546 secondary antibody (Invitrogen). Hoechest 33342 (Invitrogen) was used for counter staining. Images were captured using a Nikon 80i upright microscope.
